# Assessing the content validity of the migrant health country profile tools in Tunisia: A mixed methods study protocol

**DOI:** 10.1371/journal.pone.0352171

**Published:** 2026-07-08

**Authors:** Farah Seedat, Stella Evangelidou, Abdedayem Khelifi, Taha Maatoug, Anissa Ouahchi, Wejdene Mansour, Ali Mtiraoui, Mohamed Douagi, Reem Bouarrouj, Carmen Urbiztondo, Eman Elafef, Hassan Edries, Oumnia Bouaddi, Dominik Zenner, Kolitha Wickramage, Sally Hargreaves, the MENA Migrant Health Working Group, Ana Requena-Méndez

**Affiliations:** 1 The Migrant Health Research Group, City St George’s University of London, London, United Kingdom; 2 The Migrant Health Research Group, Barcelona Institute for Global Health (ISGlobal), Barcelona, Spain; 3 Office Nationale de la Famille et de la Population, Tunis, Tunisia; 4 Faculty of Medicine of Sousse, University of Sousse, Sousse, Tunisia; 5 Blue Nile National Institute for Communicable Diseases, University of Gezira, Wad Madani, Gezira, Sudan; 6 Department of Public Health and Clinical Research, Mohammed VI Center for Research and Innovation, Rabat, Morocco; 7 Mohammed VI University of Sciences and Health, Mohammed VI International School of Public Health, Casablanca, Morocco; 8 Wolfson Institute of Population Health, Queen Mary University of London, London, England; 9 Migration Health and Development Research Initiative (MHADRI), Colombo, Sri Lanka; 10 Department of Medicine Solna, Karolinska Institutet, Solna, Sweden; 11 Centro de Investigación Biomédica en Red de Enfermedades Infecciosas, Madrid, Spain; 12 CIBERINFEC, Barcelona, Spain; Access Alliance Multicultural Health and Community Services: Access Alliance, CANADA

## Abstract

**Introduction:**

Human migration is a historical constant, yet integrating migrant populations into health policy and systems remains a challenge. As highlighted by UN agencies and The Lancet Migration Commission, a key issue exacerbating inequities is the lack of data on disease indicators and healthcare access for migrants, with existing datasets often fragmented. Our transdisciplinary consortium co-developed the Migrant Health Country Profile tools (MHCP-t) – an innovative package of three survey tools that nationally map migrant health data sources (*MHCP-t: Data tool*), document migrant health policies and the integration of migrants in policies (*MHCP-t: Policy tool*), and the integration of migrants into healthcare provision (*MHCP-t: Provision tool*) for key diseases. This protocol describes a mixed-methods validation study investigating the content validity, feasibility, and acceptability of the MHCP-t in Tunisia.

**Methods:**

We will recruit a purposive sample of 40–50 national experts in migration health to complete one of the three MHCP-t according to their expertise. Two subsequent surveys will evaluate: 1) item-level content validity (relevance, comprehensiveness, and comprehensibility for individual items) for the *MHCP-t: Policy* and *MHCP-t: Provision*, where items function as indicators of the extent to which migrants are integrated into health policies and service provision and therefore require direct assessment at the item level, and 2) tool-level validity, feasibility, and acceptability for all three tools, including the *MHCP-t: Data* tool, where the primary objective is to assess the comprehensiveness and relevance of the inventory as a whole rather than the validity of individual items as indicators. This will be followed by focus group discussions with a nested sub-sample of approximately 10–15 participants from the initial survey to provide qualitative depth of participants’ perspectives. Item-level content validity will be analysed using the Item/Scale Content Validity Indices and Krippendorff’s Alpha (α). To assess the level of agreement, the interquartile range will be calculated for each item based on Likert scale ratings (1–5). The second survey will be analysed using descriptive statistics while qualitative data will undergo thematic analysis.

**Discussion:**

Findings will be disseminated via peer-reviewed publications, conferences, and other formats relevant to different stakeholders. By integrating these findings, the study will determine the tools’ appropriateness for mapping migrant health data sources, policies, and service provision, informing their potential for wider national rollout across the MENA region and other contexts.

## Introduction

While human migration is a historical constant, the integration of migrant populations into health policy and systems remains a challenge. Migrants are defined as people who move away from their place of usual residence, whether within a country or across an international border, temporarily or permanently, and for a variety of reasons, which includes refugees and undocumented individuals. [1] Some of these migrants live in precarious conditions, with limited healthcare access, leading to worse health outcomes than non-migrants. [2–5] A key issue exacerbating health inequalities is the lack of data on disease indicators and healthcare access for migrants, with existing datasets often fragmented. [3,6,7] This makes it hard to quantify migrant health outcomes, disease prevalence, and vaccination coverage, hindering the design of effective interventions to transform their wellbeing.

Organisations like the United Nations Department of Economic and Social Affairs (UNDESA) and the International Organization for Migration (IOM), provide useful data on migration stocks and flows, but they lack the depth and specificity to migrant health indicators. [8] For example, IOM’s Migration Governance indicators (MGI) covers migration governance, [9,10] while another tool, the Migrant Integration Policy Index (MIPEX), only measures how well a country’s policies integrate migrants. Conversely, data from health sources including WHO are more health-relevant, but they are often not disaggregated by migration, limiting their utility for addressing migrant health. [11] Indeed, WHO, IOM and *The Lancet Migration Commission* have identified a global gap in high-quality and policy-relevant migrant health data, limiting the capacity of health systems to respond effectively. [3,7,12] They highlight that little to no health-related Sustainable Development Goals (SDG) data is disaggregated by migratory status, [13] and emphasise the urgent need for migrant data to be integrated into national health information systems to improve sustainable policy implementation. [14] The COVID-19 pandemic underscored the importance of including vulnerable populations in preparedness, resilience and response plans against global health threats. [11,15,16]

**Fig 1:**
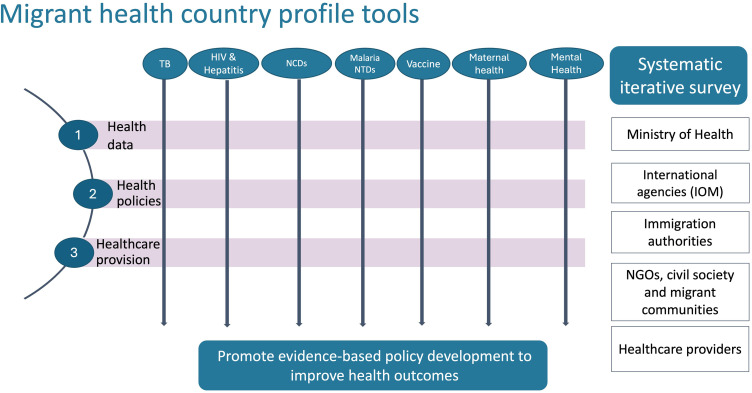
The Migrant Health Country Profile-tool schematic.

Our transdisciplinary consortium, comprising academics, policymakers and migrant communities has co-developed the Migrant Health Country Profile tools (MHCP-t) in the Middle East and North Africa. The MHCP-t, hosted on RedCap platform, is an innovative package of 3 survey tools that nationally map 1) migrant health data sources (*MHCP-t: Data*), 2) migrant health policies (*MHCP-t: Policy*) and 3) migrant healthcare provision (*MHCP-t: Provision*) for key diseases, including infectious diseases, non-communicable diseases (NCDs), mental health, and maternal and child health (Fig 1). [17–19] Integrating information from multiple sectors such as government, hospitals, and Non-Governmental Organisations (NGOs), the MHCP-t systematically assesses gaps in health data and care within a country. This serves as a crucial first step to strengthen data-informed approaches to facilitate the integration of migrants in health systems, reduce health inequities, improve migrant health outcomes, and inform real-time interventions.

The MHCP-t comprises three distinct iterative surveys with detailed questions that are designed for respondents with specific roles and expertise in migration health (see Table 1 for respondent profiles).

**Table 1:** Respondent profiles for the Migrant Health Country Profile tools (MHCP-t).

Tool	Respondents
**MHCP-t: DATA**	• **Any individuals working with or having access to data related to migrant health** • All infectious and non-infectious disease areas required – at least one of each
**MHCP-t: POLICY**	• **Top level policymakers** • These individuals should hold administrative or policy-related roles in migrant health (e.g., civil servants, policymakers, regional government officials, healthcare administrators) and key disease areas or in specific diseases programmes (e.g., HIV or tuberculosis national programmes)
**MHCP-t: PROVISION**	• **Grass-roots level professionals** • These individuals should work directly with migrants at the community or service-delivery level (e.g., clinicians, NGO health workers, community health staff).

1. **MHCP-t: Data** maps data sources for migrant health and is completed by professionals working with migration health data across sectors (e.g., government, NGOs, health systems). Respondents may possess broad expertise or specialise in specific health topics. The tool comprises three sections: respondent information, general dataset characteristics, and detailed health areas (including infectious diseases, NCDs, mental health, and maternal/child health). For each health area where a dataset contains relevant disease information, a sub-section activates to capture whether detailed, disease-specific indicators are collected.
2. **MHCP-t: Policy** documents policies for migrant health and targets high-level policymakers and administrators in migration health, including civil servants, government officials, and healthcare system leaders responsible for shaping policies and programmes. The tool comprises seven sections: respondent information, followed by five sections related to general health – international conventions, regional conventions, national and local frameworks, the integration of migrants into healthcare entitlement frameworks, and measures of policy implementation and change, and finally, policies/clinical guidelines/protocols related to specific key health areas completed depending on area of expertise.
3. **MHCP-t: Provision** explores the integration of migrants into healthcare provision and is intended for frontline workers who engage directly with migrant communities, such as clinicians, NGO staff, and community health workers involved in service delivery. The tool comprises a respondent information section, followed by five sections on general health – entitlement, information and orientation services, health education and health promotion, interpretation services, and deportations, and finally disease-specific healthcare provision, again dependent on area of expertise.

(See S1–S3 Figs for screenshots of the tool.)

Since some questions are tailored to particular health areas, respondents across all tools should possess a mix of disease expertise, including infectious diseases, NCDs, maternal and child health, and mental health. Questions dynamically adapt to align with the respondent’s background. The tool is available in Arabic, English, and French.

The MHCP-t surveys were co-developed in a stepwise process that integrated a process evaluation model with active involvement and engagement of multilevel stakeholders. The full protocol and the results of the process evaluation have been described elsewhere, [18] but are briefly summarised here. We created an in-country MHCP-t working group and a wider international MHCP-t working group to coordinate these steps. First, systematic reviews and qualitative field activities were conducted in different regions in Morocco, Tunisia, and Egypt with migrants (n = 50 per region), migrant community leaders (n = 20 per region) and professionals working with them (n = 20 per region). [19–23] This resulted in the generation of the first draft of the indicators for the MHCP-t, which were then reviewed by national and international external experts using the Nominal Group Technique. The external experts were professionals with experience on indicators for each disease area as well as some who had expertise on migration indicators and survey design in general. The national experts were stakeholders who work on migration health across different sectors and disease areas within each country. The revised indicators were entered into an electronic data capture system (RedCap) and the tool was pilot-tested with 20 migrant health experts in Tunisia by applying a mixed-methods process evaluation to examine its relevance, comprehensiveness, comprehensibility and other practical issues, such as completion time and ease of responding.

With the initial version of the MHCP-t now complete, our next step involves evaluating their content validity through independent assessment. Content validity examines whether an instrument sufficiently captures the full domain of the construct it aims to measure, ensuring the included items properly represent all relevant aspects. [24,25] This validation process will involve both item-level (question-level) content validity and tool-level (or scale-level) content validity. Specifically, item- and tool-level content validity will be assessed for the entitlement and measures of policy implementation and change sections of *MHCP-t: Policy tool* as well as for the complete *MHCP-t: Provision tool*. For the remaining sections of the *MHCP-t: Policy tool* and the complete *MHCP-t: Data* tool, content validity will be assessed only at the tool-level. These domains fundamentally differ as they serve primarily as mapping exercises rather than evaluative instruments. All three tools will undergo assessment of feasibility and acceptability.

## Research questions and objectives

The aim of this study is to investigate the content validity, feasibility, and acceptability of the Migrant Health Policy Country Profile Tools when implemented in Tunisia.

### Objectives

1. The primary objective of this study is to evaluate the content validity of the three Migrant Health Country Profile Tools: (1) MHCP-t: Data, (2) MHCP-t: Policy, and (3) MHCP-t: Provision. This will involve tool-level content validity of all three tools and item-level content validity for the entitlement and measures of policy implementation and change sections of the MHCP-t: Policy tool and the MHCP-t Provision tool.
2. As a secondary objective, we will assess the feasibility and acceptability of implementing all three tools in the Migrant Health Country Profile package.

## Methods and analysis

### Study design

This mixed-methods study employs a structured approach to evaluate content validity, feasibility, and acceptability of the MHCP-t and follows published and peer-reviewed guidelines for tool validation. [24,25] The investigation prioritises content validation through systematic examination of three fundamental domains: relevance, comprehensiveness, and comprehensibility.

The study implements a convergent parallel mixed-methods design combining qualitative and quantitative approaches. Focus group discussions with respondents will yield in-depth qualitative data regarding user experiences and perceptions of the tools. Concurrently, structured surveys will generate quantitative metrics assessing various performance characteristics. This dual-method approach facilitates comprehensive identification of potential limitations, including conceptual gaps in content coverage, ambiguities in item formulation, and practical challenges in administration protocols. The integrated analysis of both qualitative and quantitative findings will provide an evidence base for subsequent refinements, ultimately enhancing both the scientific validity and practical utility of the MHCP-t instruments in real-world settings.

### Sampling and participant recruitment

Guidelines for sample sizes for validation studies vary widely. For qualitative content validation, studies typically require 7–10 participants, however, data saturation is more important, whereas survey-based approaches generally necessitate 50–100 respondents to ensure adequate statistical power. [25] Balancing sample size requirements against the limited pool of individuals with appropriate expertise in migrant health policy and health system integration, we will employ purposive sampling to recruit approximately 40–50 eligible participants who meet the criteria outlined in Table 1.

To ensure comprehensive representation, we will stratify recruitment according to four key dimensions: (i) geographic distribution across all regions of Tunisia; (ii) professional specialisation across data, policy, and healthcare delivery domains; (iii) balanced expertise in all health condition/ disease areas (with equal representation for each category); (iv) and demographic diversity. An essential exclusion criterion will be prior involvement in MHCP-t development phases to maintain evaluator independence. This approach ensures that our sample reflects the full spectrum of relevant expertise while maintaining methodological rigor in the validation process.

The MHCP-t Expert Working Group in Tunisia will initiate contact with potential respondents, providing comprehensive study information and extending formal invitations. Upon agreement to participate, professionals will receive a unique, secure RedCap link to access the MHCP-t platform. The first page of the digital tool will present the written informed consent form, which participants must complete before proceeding.

### Tool administration and data collection

The national research teams will first conduct comprehensive training sessions delivered by two qualified members. These sessions will guide participants through the proper use of the MHCP-t platform and validation questionnaires, while also providing opportunities to address any technical or procedural questions. Following this orientation, participants will have a two-week window to complete both the assessment tools and accompanying validation instruments through the secure RedCap interface at their convenience in the comfort of their homes. Based on the process evaluation, we anticipate that completion of one MHCP-t and the associated validation questionnaires will take approximately five hours in total; however, participants will be able to complete the tool and questionnaires in stages, with the flexibility to return to them over time. To encourage completion and reduce respondent burden and fatigue, participants will receive a gift voucher upon completion of the tool. In addition, as participants are contacts identified through members of the expert working group network, we will follow them up to monitor progress and to offer support as needed during completion of the tool and validation questionnaires.

### Validation, acceptability, and feasibility questionnaires

Upon completing the relevant MHCP-t, participants of the *MHCP-t: Policy tool* and *MHCP-t: Provision tool* will encounter two structured validation questionnaires administered through the RedCap platform while participants of the *MHCP-t: Data tool* will only receive one. The first questionnaire focuses on item-specific content validity assessment, asking respondents to evaluate each question’s quality across the dimensions of comprehensibility, relevance, and capturability. Using a 5-point Likert scale ranging from “Very low quality” (1) to “Exceptional quality” (5), participants will rate each item. Those providing ratings of 1 or 2 will be required to submit qualitative explanations for their low evaluations.

This granular validation approach will be applied uniformly throughout the entire *MHCP-t: Provision tool* and will not be applied for the *MHCP-t: Data tool*. For the *MHCP-t: Policy tool*, evaluation will be restricted to the measures of policy implementation and change and entitlement sections. Items within these sections as well as those in the *MHCP-t: Provision tool* function as indicators of the extent to which migrants are integrated into health policies and healthcare service provision and therefore require direct item-level assessment. The remaining sections of the *MHCP-t: Policy tool* together with the *MHCP-t: Data tool* serve primarily as mapping exercises rather than evaluative instruments; consequently item-level validity is not required for these sections.

The second questionnaire adopts a holistic approach, assessing tool-level content validity as well as feasibility, and acceptability across all three tools in the MHCP-t package. This comprehensive evaluation incorporates the standardised questions detailed in Box 1, allowing for systematic comparison across tool types and user groups. Three questions (3, 4, and 7) assess tool-level content validity of each tool, specifically addressing the relevance, comprehensiveness, and comprehensibility of each tool as a whole. This assessment is conducted for all three tools, including the *MHCP-t: Data tool* to determine whether the overall tool adequately captures the intended purpose. The remaining questions assess feasibility and acceptability. Respondents will be asked to rate each question on a 4-point Likert scale with the exception of question 4 which has a binary response of “yes” or “no”.

### Focus group discussions

Alongside the quantitative assessments, the validation study will include a focus group discussion with 5–10 participants for each tool, onsite or off-site as convenient. These semi-structured discussions will explore four key themes: the tools’ perceived strengths, identified areas for improvement, potential applications in daily practice, and feasibility of integration into routine reporting systems. Trained researchers will document these sessions through detailed field notes to ensure comprehensive data capture for subsequent analysis.

### Demographic profiling

All participants engaged in the study will provide essential sociodemographic information to enable proper characterisation of the respondent pool. This will include standard demographic variables, professional details (occupation, position, and institutional affiliation), geographic coverage parameters, and specialised expertise areas – with particular attention to distinguishing between infectious disease and NCD specialisation.

### Data analysis

#### Quantitative data from surveys.

The psychometric evaluation will employ three robust validation metrics to systematically assess the content validity of the items of the *MHCP-t: Policy* and relevant sections of the *MHCP-t: Provision* tools. First, the Item Content Validity Index (I-CVI) will quantify the proportion of respondents endorsing each item as either “high quality” (4) or “exceptional quality” (5). Items failing to meet the established threshold of 0.78 will be systematically reviewed for potential modification or elimination. Second, the Scale Content Validity Index (S-CVI/Ave) will provide a comprehensive evaluation of the entire instrument by averaging I-CVI scores across all items, with benchmarks set at ≥0.90 for excellent validity, 0.80–0.89 for good validity requiring minor revisions, and <0.80 indicating need for substantial refinement. Third, we will compute Krippendorff’s Alpha (α) to evaluate inter-rater reliability, interpreting coefficients as: strong agreement (α ≥ 0.80), moderate agreement (0.67 ≤ α < 0.80), and weak agreement (α < 0.67). All statistical computations will be performed in STATA 18 using specialised packages (*krippalpha, kappaetc*) to ensure methodological precision. [24]

The second questionnaire (Box 1) will be subjected to detailed descriptive statistical analysis for all three MHCP-t, including the MHCP-t: Data tool, which will only have this part of the analysis for the tool-level validity, feasibility, and acceptability. We will compute measures of central tendency (mean, median) and dispersion (standard deviation, range) to characterise response patterns across all participants and items. These analyses will provide empirical evidence regarding the tool’s overall validity, feasibility, and acceptability.

All quantitative analyses will be performed using STATA 18 statistical software.

**Box 1. Survey for assessing the tool-level content validity, feasibility, and acceptability of the Migrant Health Country Profile tools**

Practical issues

1. How do you rate the REDCap platform?

Very good Good Fair Poor

2. To what extent do you think the time allocated for completing the tool was sufficient?

A lot Somewhat  Little Not at all Relevance

3. To what extent do the questions/items/indicators cover all the relevant areas on Migrant Health?

Very good Good Fair Poor

Comprehensiveness

4. Are there any questions/items/indicators in the tool that you think are lacking important elements regarding Migrant Health?

Yes  No

Comprehensibility

5. How do you rate the manual provided alongside the tool?

Very good Good Fair Poor

6. How do you rate the training you received alongside the tool?

Very good Good Fair Poor

7. To what extent are the questions/items/indicators clear and easy to understand?

Very good Good Fair Poor

Applicability

8. How would you rate the applicability of the tool in your settings?

Very good Good Fair Poor

Acceptability

9. To what extent would the tool be useful in your context and accepted in your sector as a public health intervention for identifying gaps in migrant health data collection by all relevant stakeholders (such as policymakers, migrants, health professionals, NGOs, etc)?

Very good Good Fair Poor

#### Qualitative thematic analysis.

Focus group discussion field notes and open-ended survey responses will undergo rigorous thematic analysis per tool using NVivo 14. Our analytical approach will combine:

1. Deductive coding based on a priori categories aligned with our validation framework, including the elements shown in Box 1 – practical issues, relevance, comprehensiveness, comprehensibility, applicability, and acceptability.
2. Inductive coding to capture emergent themes from participant narratives
3. Constant reflexive analysis to identify patterns across participant subgroups

The coding process will involve multiple iterations to develop a comprehensive codebook, followed by thematic mapping to identify relationships between categories. Representative anonymised quotations will be selected to illustrate each thematic finding, maintaining methodological transparency while protecting participant confidentiality.

### Patient and public involvement

This project has included a participatory research approach. Members of the Tunisian working group and the wider MENA Migrant Health Working Group, including clinicians, policymakers, UN agencies, NGOs, and those with lived experiences of migration have been involved in the design of this protocol.

### Ethics

The study has been approved by the ethics committees of the Hospital Clinic, Barcelona, Spain (HCB/2022/0655) and the University of Sousse, Medicine Faculty, Tunisia (CEFMS 157/2023).

Members of the research team will discuss the nature of the research and our study objectives with participants. The first page of the digital tool will present the written informed consent form, which participants must complete before proceeding. As with the entire survey, this page will also be available in Arabic, English, and French languages. The opportunity will be given for participants to ask any questions about the scope of the research or their rights as participants. The confidentiality of the data will be also highlighted.

### Status and timeline of study

We are currently in the participant recruitment phase, which started on 19 September 2025 and should be completed by the first week of February 2026. Training and data collection will then commence during February and March 2026, with results expected in July 2026.

### Inclusivity in global research

Additional information regarding the ethical, cultural, and scientific considerations specific to inclusivity in global research is included in the Supporting Information (S4 Checklist).

## Discussion

### Strengths and limitations

A key strength of this study is its mixed-methods approach, generating robust quantitative psychometric evidence alongside rich qualitative insights to inform tool refinement in a given context. The protocol was developed, in consultation with an interdisciplinary, international MHCP-t working group, including policymakers, health professionals, UN agencies, methodologists, NGOs, and individuals with lived migration experiences. The sample size will be as large as feasible within the study context, aiming for representativeness across specialties, demographics, and geography. We anticipate approximately 40–50 participants per tool, which will be just around the minimum standard for such studies. [25] However, there is a risk that the time required to complete the tool and validation survey may be insufficient, potentially leading to respondent overload and a low number of feasible returns. To mitigate this, we will provide in-depth training and facilitate deep engagement with respondents, led by the credible leadership and members of the national working group, we will follow participants up, and compensate them with a gift voucher. Finally, while item-level content validity will be assessed for the *MHCP-t: Policy* and *MHCP-t: Provision* tools, this is not feasible for the *MHCP-t: Data* tool, which inventories sources rather than scalable constructs. However, the overall content validity of the *MHCP-t: Data* tool will be assessed, providing the necessary evidence for its validation and refinement.

### Dissemination plans

We will pursue a comprehensive dissemination strategy to ensure our findings achieve maximum policy, academic and societal translational impact. Given the international significance of this work, we will submit a manuscript to high-quality, peer-reviewed journals to subject our research to rigorous scholarly scrutiny while reaching a global audience. We will complement this by presenting our results at relevant conferences nationally, regionally, and internationally through both oral and poster presentations.

Our engagement with policymakers will include a formal report to Tunisia’s Ministry of Health, presented in a format designed to directly inform policy development. Concurrently, we will maintain an open-access approach by publishing key findings on the MENA Migrant Health project website (http://www.menamigranthealth.org/), ensuring accessibility for diverse stakeholders.

Recognising the vital role of community engagement, we will develop tailored knowledge exchange materials for civil society organisations, migrant support groups, UN agencies and NGOs across the MENA region. Through our established networks, we will facilitate workshops and produce policy briefs to enable these partners to utilise the evidence effectively in their advocacy work and service improvement initiatives.

The validated tools will provide:

- Robust assessment of migrant health data quality, healthcare provision, and policy implementation.
- Identification of critical gaps in migrant-related data, policy frameworks, and service provision
- Evidence to transform migration health surveillance and strengthen health system responses for migrant populations.
- A standardised approach enabling cross-national comparisons in the region.

This will directly contribute to improving health outcomes for migrant communities in Tunisia while establishing transferable methodologies for the wider MENA region and globally. The validation process ensures the tools’ accuracy for both research and policy advocacy purposes, enhancing their long-term utility. By systematically identifying health system gaps and proposing evidence-based solutions, the tools will inform more equitable health policies at local, national, and regional levels.

### Strengths and limitations of the study

- A robust, transparent validation methodology yielding both quantitative psychometric evidence and rich qualitative insights.
- A multidisciplinary international team, incorporating expertise in migrant health, policy, and methodology alongside lived experience.
- Potential limitation in sample size due to the highly specialised expert pool required by the tools’ purpose.

Item-level content validity analysis is not feasible for the *MHCP-t: Data tool*, as it inventories data sources rather than scalable constructs.

## Supporting information

S1 Fig Screenshot examples of the Migrant Health Country Profile Tool: Data. (TIFF)

S2 Fig Screenshot examples of the Migrant Health Country Profile Tool: Policy. (TIFF)

S3 Fig Screenshot examples of the Migrant Health Country Profile Tool: Provision. (TIFF)

S4 Checklist Inclusivity in global research questionnaire. (DOCX)
