# Supplementary figures and images for: Assessing the content validity of the migrant health country profile tools in Tunisia: A mixed methods study protocol
Source: PLoS One. 2026 Jul 8;21(7):e0352171. doi: 10.1371/journal.pone.0352171 (PMC13345276; doi:10.1371/journal.pone.0352171)

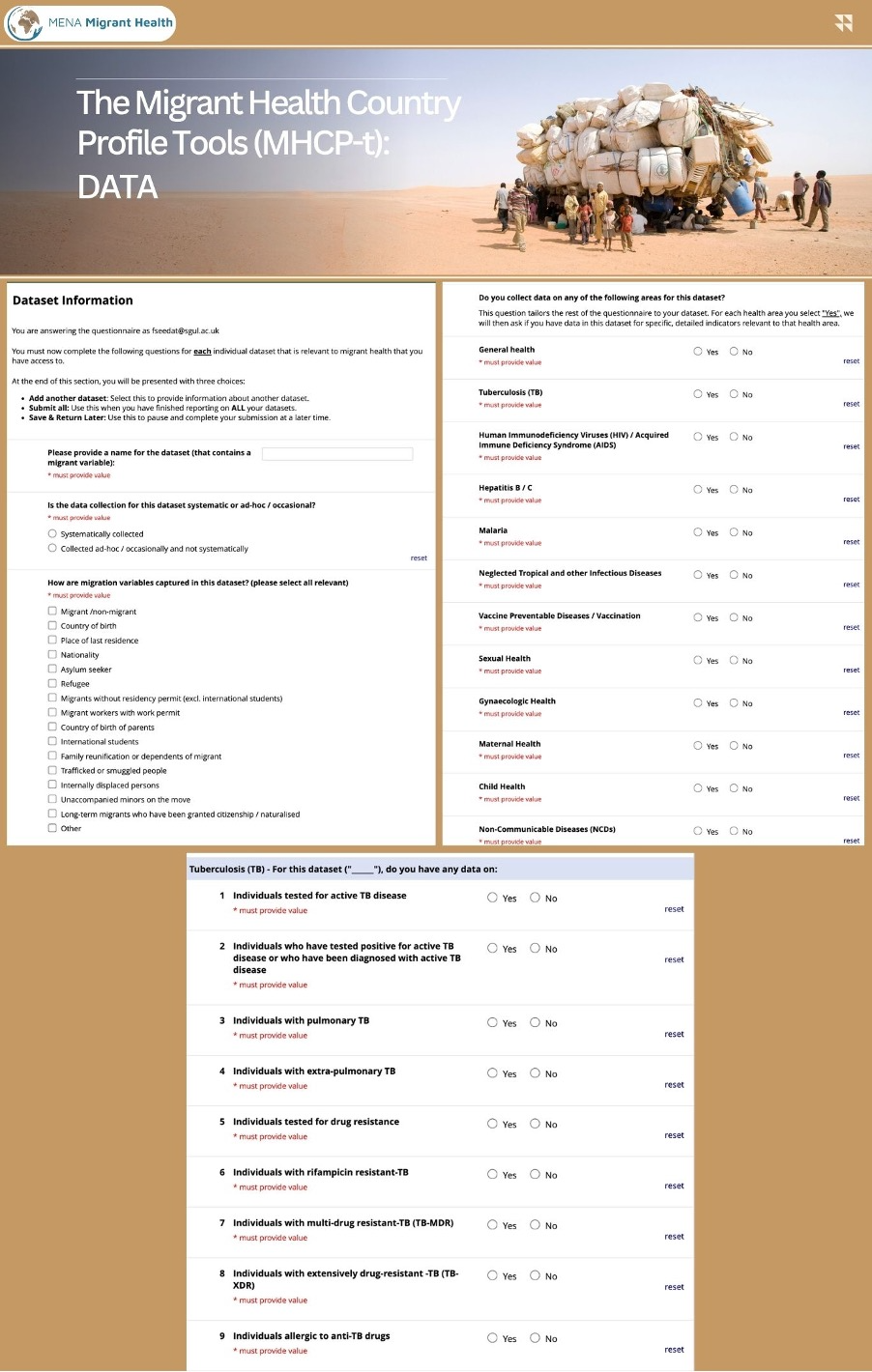

Supplement: S1 Fig — (TIFF) [file pone.0352171.s001.tiff]

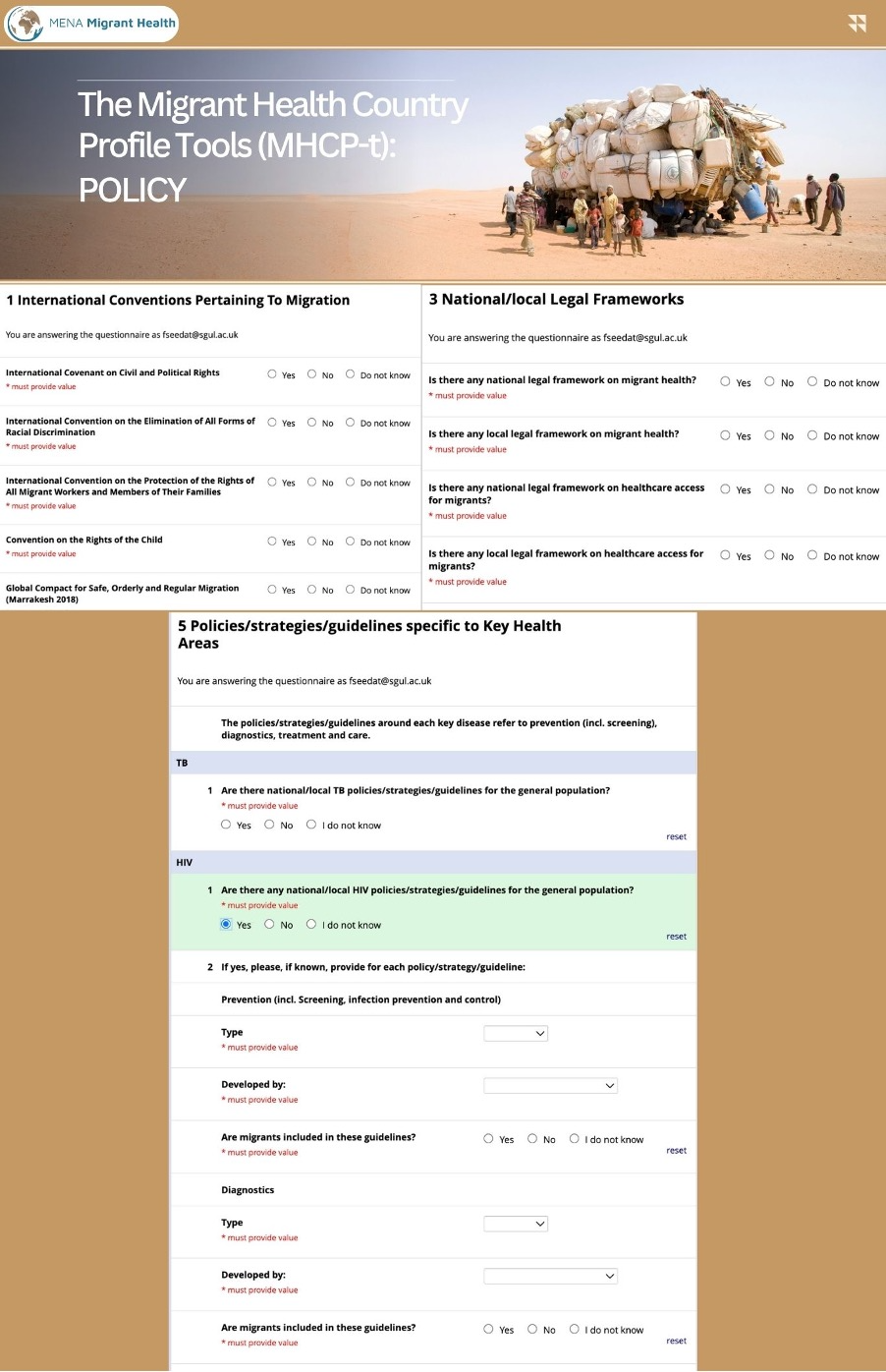

Supplement: S2 Fig — (TIFF) [file pone.0352171.s002.tiff]

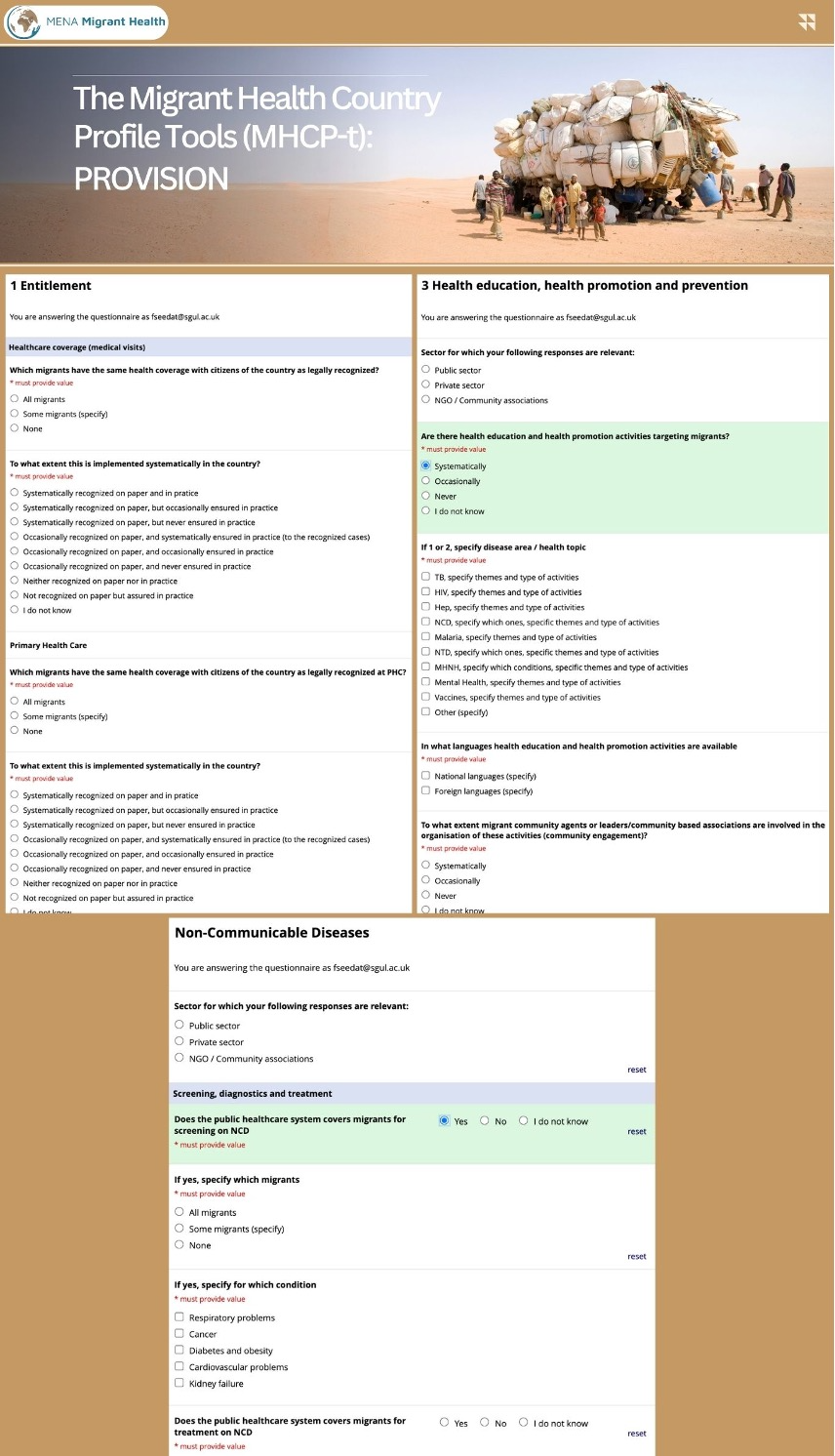

Supplement: S3 Fig — (TIFF) [file pone.0352171.s003.tiff]
