# Supplementary material for: Assessing the content validity of the migrant health country profile tools in Tunisia: A mixed methods study protocol
Source: PLoS One. 2026 Jul 8;21(7):e0352171. doi: 10.1371/journal.pone.0352171 (PMC13345276; doi:10.1371/journal.pone.0352171)
Supplement: Attachment — Submitted filename: MHCPt_Validation_PLOSONE_Response_Final.docx [file pone.0352171.s006.docx]

The Editorial Office

PLOS ONE

23 April 2026

Dear Dr Alamgir,

Thank you for the opportunity to revise our manuscript **“Assessing the content validity of the Migrant Health Country Profile Tools in Tunisia: A mixed methods study protocol” (PONE-D-25-69071R1)**. In response to your valuable feedback, we have carefully examined the reviewers' and editors’ comments and have effectively addressed them as outlined in the response table below and in the marked and clean manuscript document.

Our response demonstrates our commitment to collaborating with PLOS ONE and aligning our manuscript with the journal’s high standards. We are happy to make any further changes, if required.

Thank you for your time, support, and consideration.

Sincerely,

Farah Seedat & Ana Requena Mendez

**Assessing the content validity of the Migrant Health Country Profile Tools in Tunisia: A mixed methods study protocol**

**PONE-D-25-69071R1 manuscript resubmission: Response to reviewer comments**

| **No** | **Comment** | **Response** |
| --- | --- | --- |
| **Reviewer 1** | | |
| 1 | Clarity on the Validation of the MHCP-t: Data Tool: The manuscript's handling of the MHCP-t: Data tool's validation is inconsistent and potentially a conceptual flaw.  The Methods section states that content validity for the Data tool will only be assessed at the "scale level" using the second questionnaire. The Abstract and Objectives imply a full content validity assessment for "all three tools."  The justification that it "inventories sources rather than scalable constructs" (Line 63, 334) is a critical distinction. However, the protocol then proposes to assess its "overall content validity" (Line 335) using questions about relevance and comprehensiveness from Box 1.  Action Required: The authors must more clearly define what "content validity" means for an inventory tool versus an evaluative scale. Is the goal to ensure the list of data sources is comprehensive and relevant, rather than validating a set of scaled items? The distinction between "item-level" and "scale-level" validity for this tool should be explicitly stated and consistently applied throughout the manuscript, particularly in the Abstract, Objectives, and Data Analysis sections. The authors should consider if a different term, such as "utility" or "comprehensiveness," might be more accurate for the Data tool's assessment to avoid conflating it with the psychometric validation of the other two tools. | Thank you for this important comment, which highlights the need for greater conceptual clarity in validating the MHCP t: Data tool. We agree that the distinction between validating an inventory tool versus an evaluative scale must be articulated more explicitly and applied consistently throughout the manuscript.  Indeed, the MHCP t: Data tool is designed as an inventory of existing data sources, not as an evaluative scale. Its individual entries correspond to external entities (e.g., registries, surveillance systems, and administrative datasets), and the primary purpose is to determine what these data sources cover, rather than to produce scaled items. Consequently, item-level content validity, which assesses whether individual items are relevant indicators of an underlying construct, is not conceptually applicable to the Data tool. Instead, validity for this tool is addressed at the tool level, focusing on whether the tool as a whole is relevant, comprehensive, and fit for the purpose of mapping the national data landscape for migrant health indicators. In this context, our use of the term “content validity” refers to the adequacy of coverage and relevance of the inventory, not to psychometric validation of individual item indicators. To avoid conflation with scale-based validation approaches, we now explicitly frame this assessment as tool-level validity, encompassing the relevance, comprehensiveness, feasibility, and acceptability of the entire tool.  In contrast, the MHCP t: Policy tool and relevant parts of the MHCP t: Provision tool operationalise defined constructs related to how well migrants are integrated into policy environments and service provision. Their items function as indicators of these constructs and therefore require item-level content validity assessment to ensure relevance, clarity, and comprehensive coverage of the underlying constructs.  To address this concern, we have revised the **Abstract, Objectives, Methods, and Data Analysis** sections to consistently state this, as below:  **Abstract (page 3 lines 44-50):**  *“Two subsequent surveys will evaluate: 1) item-level content validity (relevance, comprehensiveness, and comprehensibility* ***for individual items****) for the MHCP-t: Policy and MHCP-t: Provision,* ***where items function as indicators of the extent to which migrants are integrated into health policies and service provision and therefore require direct assessment at the item level****, and 2)* ***tool-level*** *validity, feasibility, and acceptability for all three tools,* ***including the MHCP-t: Data tool, where the primary objective is to assess the comprehensiveness and relevance of the inventory as a whole rather than the validity of individual items as indicators****.”*  We have also expanded on the explanation of the tools in the Abstract (page 3 lines 38-39) to make this distinction clear:  *“Our transdisciplinary consortium co-developed the Migrant Health Country Profile tools (MHCP-t) – an innovative package of three survey tools that nationally map migrant health data sources (MHCP-t: Data tool),* ***document migrant health policies and the integration of migrants in policies (MHCP-t: Policy tool), and the integration of migrants into healthcare provision (MHCP-t: Provision tool)*** *for key diseases.”*  **Introduction:**  **Page 7 lines 124-128**  *“****MHCP-t: Policy*** *documents policies for migrant health … The tool comprises seven sections: …* ***the integration of migrants into healthcare entitlement frameworks****, and measures of policy implementation and change*.  ***MHCP-t: Provision*** *explores* ***the integration of migrants into*** *healthcare provision..”*  **Page 9 lines 164-172:**  *“This validation process will involve both item-level (question-level) content validity and tool-level (or scale-level) content validity. Specifically, item- and tool-level content validity will be assessed for the entitlement and measures of policy implementation and change sections of MHCP-t: Policy tool as well as for the complete MHCP-t: Provision tool. For the remaining sections of the MHCP-t: Policy tool and the complete MHCP-t: Data tool, content validity will be assessed only at the tool-level. These domains fundamentally differ as they serve primarily as mapping exercises rather than evaluative instruments.”*  **Objectives** (**page 9 lines 181-3**):  *“The primary objective of this study is to evaluate the content validity of the three Migrant Health Country Profile Tools: (1) MHCP-t: Data, (2) MHCP-t: Policy, and (3) MHCP-t: Provision.* ***This will involve tool-level content validity of all three tools and item-level content validity for the entitlement and measures of policy implementation and change sections of the MHCP-t: Policy tool and the MHCP-t Provision tool****.”*  **Methods**  **Pages 12-13 lines 246-275:**  *“Upon completing the relevant MHCP-t, participants* ***of the MHCP-t: Policy tool and MHCP-t: Provision tool*** *will encounter two structured validation questionnaires administered through the RedCap platform* ***while participants of the MHCP-t: Data tool will only receive one****. The first questionnaire focuses on item-specific content validity assessment, asking respondents to evaluate each question's quality across the dimensions of comprehensibility, relevance, and capturability. Using a 5-point Likert scale ranging from "Very low quality" (1) to "Exceptional quality" (5), participants will rate each item. Those providing ratings of 1 or 2 will be required to submit qualitative explanations for their low evaluations.*  *This granular validation approach will be applied uniformly throughout the entire MHCP-t: Provision tool and will not be applied for the MHCP-t: Data tool.* ***For the MHCP-t: Policy tool, evaluation will be restricted to the measures of policy implementation and change and entitlement sections. Items within these sections as well as those in the MHCP-t: Provision tool function as indicators of the extent to which migrants are integrated into health policies and healthcare service provision and therefore require direct item-level assessment. The remaining sections of the MHCP-t: Policy tool together with the MHCP-t: Data tool serve primarily as mapping exercises rather than evaluative instruments; consequently item-level validity is not required for these sections.***  *The second questionnaire adopts a holistic* ***approach****, assessing* ***tool-level*** *content validity as well as feasibility, and acceptability across all three tools in the MHCP-t package. This comprehensive evaluation incorporates the standardised questions detailed in Box 1, allowing for systematic comparison across tool types and user groups.* ***Three questions (3, 4, and 7) assess overall tool-level content validity of each tool, specifically addressing the relevance, comprehensiveness, and comprehensibility of each tool as a whole. This assessment is conducted for all three tools, including the MHCP-t: Data tool to determine whether the overall tool adequately captures the intended purpose.*** *The remaining questions assess feasibility and acceptability. Respondents will be asked to rate each question on a 4-point Likert scale with the exception of question 4 which has a binary response of “yes” or “no”.”*  **Page 15 lines 295 and 308-309:**  *“The psychometric evaluation will employ three robust validation metrics to systematically assess the content validity of* ***the items of the MHCP-t: Policy and relevant sections of the MHCP-t: Provision tools****.*  *The second questionnaire (box 1) will be subjected to detailed descriptive statistical analysis* ***for all three MHCP-t, including the MHCP-t: Data tool, which will only have this part of the analysis for the tool-level validity, feasibility, and acceptability.”*** |
| 2 | Feasibility of "Exhaustive Sampling": The manuscript proposes to recruit an "exhaustive sample" of 40-50 participants for each tool (Line 200), totaling 120-150 experts. This is an ambitious target in a specialized field within a single country.  Action Required: The authors should provide more detail on the expert pool in Tunisia. Are there existing registries, networks, or estimates of the total number of individuals who meet the inclusion criteria? Providing this context would strengthen the rationale for the sample size target and demonstrate its feasibility. If 40-50 truly represents the entire universe of eligible experts for each tool, this should be explicitly stated. If not, the term "exhaustive sampling" may be misleading, and a justification for why this number is adequate despite not being exhaustive should be provided. | Thank you for noting the ambitious nature of the proposed sampling strategy. The target of 40–50 participants per tool was selected by balancing published recommendations for sample sizes in content validity studies (e.g., COSMIN guidelines) with the highly specialised scope of the topic. This approach was discussed extensively with the MHCP‑t Expert Working Group, who advised that recruiting more than 50 participants for any single tool would not be feasible within the national context.  As no official registries or comprehensive networks of eligible experts exist, we worked closely with the MHCP‑t Expert Working Group to identify and invite individuals who meet the inclusion criteria for each tool. As you correctly note, this approach did not allow us to know for certain the total number of eligible experts nationwide. We therefore agree that purposive sampling is a more accurate description than “exhaustive sampling.”  Purposive sampling is appropriate in this context, as content validity assessment requires the inclusion of individuals with specific, in‑depth expertise relevant to the constructs being evaluated. Our sampling strategy was designed to ensure sufficient diversity of expertise across policy, service provision, and data systems, aligning with established methodological guidance for expert‑based validation studies. Indeed, in Tunisia the completion of the tools so far includes 25 participants for the MHCP-t: Data, 29 participants for MHCP-t: Policy, and 40 participants for MHCP-t: Provision.  We have revised the manuscript accordingly to reflect this clarification.  **Page 10 lines 205-211**  *“Guidelines for sample sizes for validation studies vary widely. For qualitative content validation, studies typically require 7-10 participants, however, data saturation is more important, whereas survey-based approaches generally necessitate 50-100 respondents to ensure adequate statistical power.[25]* ***Balancing sample size requirements against the limited pool of individuals with appropriate expertise in migrant health policy and health system integration, we will employ purposive sampling to recruit approximately 40-50 eligible participants who meet the criteria outlined in Table 1.****”* |
| 3 | Burden on Participants: The data collection process is substantial. Participants are asked to: 1) Complete their assigned MHCP-t (which itself is likely lengthy), 2) Complete an item-by-item validation survey for their tool, and 3) Complete a second global validation survey. This represents a significant time commitment.  Action Required: The manuscript would benefit from including an estimated completion time for each of the three MHCP-t tools and the accompanying surveys. This information is crucial for assessing the risk of respondent fatigue and low response rates, a limitation the authors themselves acknowledge. How will the study mitigate this burden beyond providing training? Will participants be compensated or incentivized for their time? | Thank you for highlighting the potential burden associated with the data collection process. Based on previous work in which 20 participants completed all three MHCP‑t tools over a total of nine hours, we anticipated that completion of a single MHCP‑t tool together with the associated item‑level and overall validation questionnaires would require a maximum of approximately five hours per participant. This estimate has been confirmed during the current phase of data collection.  To mitigate participant burden and reduce the risk of respondent fatigue, participants are able to complete the tool and validation questionnaires in stages, with the flexibility to return to them over time. In addition, participants are incentivised through the provision of a gift voucher upon completion of the tool. Members of the expert working group, who identified participants through their professional networks, also follow up individuals who had not yet completed the tool or surveys to identify any issues and to provide encouragement and support as needed.  We have incorporated this information into the manuscript (**page 12, lines 236–243**) to improve transparency regarding time commitment, burden mitigation strategies, and participant incentives:  ***“Based on the process evaluation, we anticipate that completion of one MHCP-t and the associated validation questionnaires will take approximately four hours in total; however, participants will be able to complete the tool and questionnaires in stages, with the flexibility to return to them over time. To encourage completion and reduce respondent burden and fatigue, participants will receive a gift voucher upon completion of the tool. In addition, as participants are contacts identified through members of the expert working group network, we will follow them up to monitor progress and to offer support as needed during completion of the tool and validation questionnaires.”***  We have also included this information in the discussion section (**page 19 line 371**). To mitigate this, we will provide in-depth training and facilitate deep engagement with respondents, led by the credible leadership and members of the national working group, ***we will follow participants up, and compensate them with a gift voucher.*** |
| **Reviewer 2** | | |
| 4 | The manuscript presents an interesting and potentially valuable proposal for the development of the Migrant Health Country Profile tools (MHCP-t). However, the current submission describes a protocol and proposed validation process rather than findings derived from an implemented study. At present, the manuscript outlines the planned mixed-methods approach, including expert surveys and focus group discussions, but the tools themselves have not yet been tested in practice and no empirical results are available.  Given this, the manuscript is a research proposal or study protocol than as a completed research article. While the methodological design are promising, the contribution to the literature would be significantly strengthened once the proposed surveys and focus group discussions have been conducted and the results analyzed. Empirical evidence demonstrating the tools’ content validity, feasibility, and acceptability would allow readers to better assess the practical usefulness and methodological robustness of the MHCP-t.  I would therefore recommend considering the manuscript for publication after the study has been completed, including the implementation of the surveys with national experts and the focus group discussions. Presenting the resulting data and analysis alongside the methodological framework would provide a more substantial contribution to the field and allow for a clearer evaluation of the proposed tools’ effectiveness and applicability. | Thank you for your thoughtful comments. This manuscript is intentionally submitted as a study protocol and is presented as such in line with PLOS ONE’s scope for publishing methodologically rigorous protocols that promote transparency, reproducibility, and early dissemination of complex study designs. As a protocol paper, it does not yet report empirical findings from the implementation of the validation study.  That said, the proposed methods are informed by prior piloting and process evaluation conducted during an earlier phase of this work, as noted on page 8. During this process evaluation, the MHCP‑t tools and associated procedures were tested with a small sample of participants, confirming the feasibility and appropriateness of the study design, recruitment strategy, and data collection approach.  We fully agree that empirical evidence on content validity, feasibility, and acceptability will further strengthen the contribution of this work. We therefore intend to publish the results of the surveys and focus group discussions in a subsequent peer‑reviewed manuscript once data collection and analysis are complete. The current protocol is intended to provide a transparent and detailed account of the planned validation process, thereby enabling critical appraisal of the methodology and supporting the reproducibility of future MHCP‑t validation studies in other national contexts. |
| 5 | **Journal Requirements:**  When submitting your revision, we need you to address these additional requirements.  1. Please ensure that your manuscript meets PLOS ONE's style requirements, including those for file naming. The PLOS ONE style templates can be found at  [https://journals.plos.org/plosone/s/file?id=wjVg/PLOSOne_formatting_sample_main_body.pdf](https://track.editorialmanager.com/CL0/https:%2F%2Fjournals.plos.org%2Fplosone%2Fs%2Ffile%3Fid=wjVg%2FPLOSOne_formatting_sample_main_body.pdf/1/010f019cd594043f-550e18b5-568d-462f-b725-64f320f44d86-000000/O9EGcUMwR4gnK32cRmjpn5GqP_9fHGQ83e6oneoNTcs=253) and  [https://journals.plos.org/plosone/s/file?id=ba62/PLOSOne_formatting_sample_title_authors_affiliations.pdf](https://track.editorialmanager.com/CL0/https:%2F%2Fjournals.plos.org%2Fplosone%2Fs%2Ffile%3Fid=ba62%2FPLOSOne_formatting_sample_title_authors_affiliations.pdf/1/010f019cd594043f-550e18b5-568d-462f-b725-64f320f44d86-000000/lgkHzDIcRqPGBKmZHKjmhilAe4tM6zfBQMdXHDsu-40=253) | We have done this to the best of our ability. Please let us know if anything is outstanding. |
| 6 | 2. Please include a complete copy of PLOS’ questionnaire on inclusivity in global research in your revised manuscript. Our policy for research in this area aims to improve transparency in the reporting of research performed outside of researchers’ own country or community. The policy applies to researchers who have travelled to a different country to conduct research, research with Indigenous populations or their lands, and research on cultural artefacts. The questionnaire can also be requested at the journal’s discretion for any other submissions, even if these conditions are not met.  Please find more information on the policy and a link to download a blank copy of the questionnaire here: <https://journals.plos.org/plosone/s/best-practices-in-research-reporting>. Please upload a completed version of your questionnaire as Supporting Information when you resubmit your manuscript. | We have completed the form and attached it as S4. We have also added a section in the methods about it.  ***“Inclusivity in global research***  *Additional information regarding the ethical, cultural, and scientific considerations specific to inclusivity in global research is included in the Supporting Information (S4 Checklist).”* |
| 7 | 3. We note that the grant information you provided in the ‘Funding Information’ and ‘Financial Disclosure’ sections do not match.  When you resubmit, please ensure that you provide the correct grant numbers for the awards you received for your study in the ‘Funding Information’ section. | The correct number is LCF/PR/SP21/52930003, which is in the funding information section. |
| 8 | 4. Thank you for stating the following financial disclosure:  “La Caixa, LCF/PR/SP21/52930003.”  Please state what role the funders took in the study.  If the funders had no role, please state: "The funders had no role in study design, data collection and analysis, decision to publish, or preparation of the manuscript."  If this statement is not correct you must amend it as needed.  Please include this amended Role of Funder statement in your cover letter; we will change the online submission form on your behalf. | This is true. We are happy for you to add:  The funders had no role in study design, data collection and analysis, decision to publish, or preparation of the manuscript. |
| 9 | 5. Please note that funding information should not appear in any section or other areas of your manuscript. We will only publish funding information present in the Funding Statement section of the online submission form. Please remove any funding-related text from the manuscript. | Noted, we have now removed it. |
| 10 | 6. In the online submission form you indicate that your data is not available for proprietary reasons and have provided a contact point for accessing this data. Please note that your current contact point is a co-author on this manuscript. According to our Data Policy, the contact point must not be an author on the manuscript and must be an institutional contact, ideally not an individual. Please revise your data statement to a non-author institutional point of contact, such as a data access or ethics committee, and send this to us via return email. Please also include contact information for the third party organization, and please include the full citation of where the data can be found. | Please amend the statement as follows:  *“The data from this study will be stored in a RedCap server hosted at Institut Pasteur in Tunisia. The anonymised data generated and analysed during this study will be made available upon reasonable request. Requests for data access will be considered by the Data Manager at IS Global (susana.mendez@isglobal.org) in consultation with the national expert working group in Tunisia, from whom permission must be obtained prior to sharing.”*  The expert working group consists of stakeholders in the migrant health field and are not a third-party organisation. |
| 11 | 7. Please upload a new copy of Figure 1 as the detail is not clear. Please follow the link for more information:  [https://journals.plos.org/plosone/s/figures](https://track.editorialmanager.com/CL0/https:%2F%2Fjournals.plos.org%2Fplosone%2Fs%2Ffigures/1/010f019cd594043f-550e18b5-568d-462f-b725-64f320f44d86-000000/Uj0iz3ycSk02ccEz8byE0BjNDWiMxnfZxNhH5H54yKY=253) | We have changed the resolution and resized using the very impressive [NAAS tool](https://ngplosjournals.pagemajik.ai/artanalysis). Please let us know if further changes are needed. |
| 12 | 8. We note that Supplementary Figures 1, 2, and 3 in your submission contain copyrighted images. All PLOS content is published under the Creative Commons Attribution License (CC BY 4.0), which means that the manuscript, images, and Supporting Information files will be freely available online, and any third party is permitted to access, download, copy, distribute, and use these materials in any way, even commercially, with proper attribution. For more information, see our copyright guidelines: <http://journals.plos.org/plosone/s/licenses-and-copyright>.  We require you to either (1) present written permission from the copyright holder to publish these figures specifically under the CC BY 4.0 license, or (2) remove the figures from your submission:  1. You may seek permission from the original copyright holder of Supplementary Figures 1, 2, and 3 to publish the content specifically under the CC BY 4.0 license.  We recommend that you contact the original copyright holder with the Content Permission Form (<http://journals.plos.org/plosone/s/file?id=7c09/content-permission-form.pdf>) and the following text:  “I request permission for the open-access journal PLOS ONE to publish XXX under the Creative Commons Attribution License (CCAL) CC BY 4.0 (<http://creativecommons.org/licenses/by/4.0/>). Please be aware that this license allows unrestricted use and distribution, even commercially, by third parties. Please reply and provide explicit written permission to publish XXX under a CC BY license and complete the attached form.”  Please upload the completed Content Permission Form or other proof of granted permissions as an "Other" file with your submission.  In the figure caption of the copyrighted figure, please include the following text: “Reprinted from [ref] under a CC BY license, with permission from [name of publisher], original copyright [original copyright year].”  2. If you are unable to obtain permission from the original copyright holder to publish these figures under the CC BY 4.0 license or if the copyright holder’s requirements are incompatible with the CC BY 4.0 license, please either i) remove the figure or ii) supply a replacement figure that complies with the CC BY 4.0 license. Please check copyright information on all replacement figures and update the figure caption with source information. If applicable, please specify in the figure caption text when a figure is similar but not identical to the original image and is therefore for illustrative purposes only. | The figures were designed by the authors using Canva and uploaded into the REDCap platform. However, as the screenshots depict the REDCap user interface, we understand that permission from REDCap may be required to publish images of the interface under a CC BY license. We have contacted REDCap to seek clarification and, where necessary, formal permission to include these figures in the manuscript. However, we have not yet received a response and would be happy to remove the figures if you prefer. |
| 13 | 9. Please include captions for your Supporting Information files at the end of your manuscript, and update any in-text citations to match accordingly. Please see our Supporting Information guidelines for more information: [http://journals.plos.org/plosone/s/supporting-information](http://track.editorialmanager.com/CL0/http:%2F%2Fjournals.plos.org%2Fplosone%2Fs%2Fsupporting-information/1/010f019cd594043f-550e18b5-568d-462f-b725-64f320f44d86-000000/L1Amntqtd0_ZuvFi9AmIaEleu2o5xkm_pBfUrdVmFMo=253). | We have now added this at the end of the manuscript (**pages 22-23**):  ***“Supporting information***  ***S1 Fig. Screenshot examples of the Migrant Health Country Profile Tool: Data***  ***S2 Fig. Screenshot examples of the Migrant Health Country Profile Tool: Policy***  ***S3 Fig. Screenshot examples of the Migrant Health Country Profile Tool: Provision***  ***S4. Inclusivity in global research questionnaire”*** |
| 14 | 10 If the reviewer comments include a recommendation to cite specific previously published works, please review and evaluate these publications to determine whether they are relevant and should be cited. There is no requirement to cite these works unless the editor has indicated otherwise. | This is not applicable to our peer review comments. |
