# Supplementary material for: Assessing the content validity of the migrant health country profile tools in Tunisia: A mixed methods study protocol
Source: PLoS One. 2026 Jul 8;21(7):e0352171. doi: 10.1371/journal.pone.0352171 (PMC13345276; doi:10.1371/journal.pone.0352171)
Supplement: Peer review history

# Peer review: Decision Letter 0

**DOI:** 10.1371/journal.pone.0352171.r001

**Authors:** AKM Alamgir

9 Mar 2026

PONE-D-25-69071

Assessing the content validity of the Migrant Health Country Profile Tools in Tunisia: A mixed methods study protocol

PLOS One

Dear Dr. Seedat,

Thank you for submitting your manuscript to PLOS ONE. After careful consideration, we feel that it has merit but does not fully meet PLOS ONE’s publication criteria as it currently stands. Therefore, we invite you to submit a revised version of the manuscript that addresses the points raised during the review process.

Please submit your revised manuscript by Apr 23 2026 11:59PM. If you will need more time than this to complete your revisions, please reply to this message or contact the journal office at plosone@plos.org. When you're ready to submit your revision, log on to https://www.editorialmanager.com/pone/ and select the 'Submissions Needing Revision' folder to locate your manuscript file.

Please include the following items when submitting your revised manuscript:

- A letter that responds to each point raised by the academic editor and reviewer(s). You should upload this letter as a separate file labeled 'Response to Reviewers'.
- A marked-up copy of your manuscript that highlights changes made to the original version. You should upload this as a separate file labeled 'Revised Manuscript with Track Changes'.
- An unmarked version of your revised paper without tracked changes. You should upload this as a separate file labeled 'Manuscript'.

If you would like to make changes to your financial disclosure, please include your updated statement in your cover letter. Guidelines for resubmitting your figure files are available below the reviewer comments at the end of this letter.

If applicable, we recommend that you deposit your laboratory protocols in protocols.io to enhance the reproducibility of your results. Protocols.io assigns your protocol its own identifier (DOI) so that it can be cited independently in the future. For instructions see: https://journals.plos.org/plosone/s/submission-guidelines#loc-laboratory-protocols. Additionally, PLOS ONE offers an option for publishing peer-reviewed Lab Protocol articles, which describe protocols hosted on protocols.io. Read more information on sharing protocols at https://plos.org/protocols?utm_medium=editorial-email&utm_source=authorletters&utm_campaign=protocols.

We look forward to receiving your revised manuscript.

Kind regards,

AKM Alamgir, PhD

Academic Editor

PLOS One

**Journal Requirements:**

When submitting your revision, we need you to address these additional requirements.

1. Please ensure that your manuscript meets PLOS ONE's style requirements, including those for file naming. The PLOS ONE style templates can be found at

https://journals.plos.org/plosone/s/file?id=wjVg/PLOSOne_formatting_sample_main_body.pdf and

https://journals.plos.org/plosone/s/file?id=ba62/PLOSOne_formatting_sample_title_authors_affiliations.pdf

2. Please include a complete copy of PLOS’ questionnaire on inclusivity in global research in your revised manuscript. Our policy for research in this area aims to improve transparency in the reporting of research performed outside of researchers’ own country or community. The policy applies to researchers who have travelled to a different country to conduct research, research with Indigenous populations or their lands, and research on cultural artefacts. The questionnaire can also be requested at the journal’s discretion for any other submissions, even if these conditions are not met.  Please find more information on the policy and a link to download a blank copy of the questionnaire here: https://journals.plos.org/plosone/s/best-practices-in-research-reporting. Please upload a completed version of your questionnaire as Supporting Information when you resubmit your manuscript.

3. We note that the grant information you provided in the ‘Funding Information’ and ‘Financial Disclosure’ sections do not match.

When you resubmit, please ensure that you provide the correct grant numbers for the awards you received for your study in the ‘Funding Information’ section.

4. Thank you for stating the following financial disclosure:

“La Caixa, LCF/PR/SP21/52930003.”

Please state what role the funders took in the study.  If the funders had no role, please state: "The funders had no role in study design, data collection and analysis, decision to publish, or preparation of the manuscript."

If this statement is not correct you must amend it as needed.

Please include this amended Role of Funder statement in your cover letter; we will change the online submission form on your behalf.

5. Please note that funding information should not appear in any section or other areas of your manuscript. We will only publish funding information present in the Funding Statement section of the online submission form. Please remove any funding-related text from the manuscript.

6. In the online submission form you indicate that your data is not available for proprietary reasons and have provided a contact point for accessing this data. Please note that your current contact point is a co-author on this manuscript. According to our Data Policy, the contact point must not be an author on the manuscript and must be an institutional contact, ideally not an individual. Please revise your data statement to a non-author institutional point of contact, such as a data access or ethics committee, and send this to us via return email. Please also include contact information for the third party organization, and please include the full citation of where the data can be found.

7. Please upload a new copy of Figure 1 as the detail is not clear. Please follow the link for more information:  https://journals.plos.org/plosone/s/figures

8. We note that Supplementary Figures 1, 2, and 3 in your submission contain copyrighted images. All PLOS content is published under the Creative Commons Attribution License (CC BY 4.0), which means that the manuscript, images, and Supporting Information files will be freely available online, and any third party is permitted to access, download, copy, distribute, and use these materials in any way, even commercially, with proper attribution. For more information, see our copyright guidelines: http://journals.plos.org/plosone/s/licenses-and-copyright.

We require you to either (1) present written permission from the copyright holder to publish these figures specifically under the CC BY 4.0 license, or (2) remove the figures from your submission:

1. You may seek permission from the original copyright holder of Supplementary Figures 1, 2, and 3 to publish the content specifically under the CC BY 4.0 license.

We recommend that you contact the original copyright holder with the Content Permission Form (http://journals.plos.org/plosone/s/file?id=7c09/content-permission-form.pdf) and the following text:

“I request permission for the open-access journal PLOS ONE to publish XXX under the Creative Commons Attribution License (CCAL) CC BY 4.0 (http://creativecommons.org/licenses/by/4.0/). Please be aware that this license allows unrestricted use and distribution, even commercially, by third parties. Please reply and provide explicit written permission to publish XXX under a CC BY license and complete the attached form.”

Please upload the completed Content Permission Form or other proof of granted permissions as an "Other" file with your submission.

In the figure caption of the copyrighted figure, please include the following text: “Reprinted from [ref] under a CC BY license, with permission from [name of publisher], original copyright [original copyright year].”

2. If you are unable to obtain permission from the original copyright holder to publish these figures under the CC BY 4.0 license or if the copyright holder’s requirements are incompatible with the CC BY 4.0 license, please either i) remove the figure or ii) supply a replacement figure that complies with the CC BY 4.0 license. Please check copyright information on all replacement figures and update the figure caption with source information. If applicable, please specify in the figure caption text when a figure is similar but not identical to the original image and is therefore for illustrative purposes only.

9. Please include captions for your Supporting Information files at the end of your manuscript, and update any in-text citations to match accordingly. Please see our Supporting Information guidelines for more information: http://journals.plos.org/plosone/s/supporting-information.

10 If the reviewer comments include a recommendation to cite specific previously published works, please review and evaluate these publications to determine whether they are relevant and should be cited. There is no requirement to cite these works unless the editor has indicated otherwise.

[Note: HTML markup is below. Please do not edit.]

Reviewers' comments:

Reviewer's Responses to Questions

**Comments to the Author**

1. Does the manuscript provide a valid rationale for the proposed study, with clearly identified and justified research questions?

The research question outlined is expected to address a valid academic problem or topic and contribute to the base of knowledge in the field.

Reviewer #1: Yes

Reviewer #2: Yes

**********

2. Is the protocol technically sound and planned in a manner that will lead to a meaningful outcome and allow testing the stated hypotheses?

The manuscript should describe the methods in sufficient detail to prevent undisclosed flexibility in the experimental procedure or analysis pipeline, including sufficient outcome-neutral conditions (e.g. necessary controls, absence of floor or ceiling effects) to test the proposed hypotheses and a statistical power analysis where applicable. As there may be aspects of the methodology and analysis which can only be refined once the work is undertaken, authors should outline potential assumptions and explicitly describe what aspects of the proposed analyses, if any, are exploratory.

Reviewer #1: Yes

Reviewer #2: Partly

**********

3. Is the methodology feasible and described in sufficient detail to allow the work to be replicable?

Descriptions of methods and materials in the protocol should be reported in sufficient detail for another researcher to reproduce all experiments and analyses. The protocol should describe the appropriate controls, sample size calculations, and replication needed to ensure that the data are robust and reproducible.

Reviewer #1: Yes

Reviewer #2: No

**********

4. Have the authors described where all data underlying the findings will be made available when the study is complete?

The PLOS Data policy requires authors to make all data underlying the findings described in their manuscript fully available without restriction, with rare exception, at the time of publication. The data should be provided as part of the manuscript or its supporting information, or deposited to a public repository. For example, in addition to summary statistics, the data points behind means, medians and variance measures should be available. If there are restrictions on publicly sharing data—e.g. participant privacy or use of data from a third party—those must be specified.

Reviewer #1: Yes

Reviewer #2: Yes

**********

5. Is the manuscript presented in an intelligible fashion and written in standard English?

PLOS ONE does not copyedit accepted manuscripts, so the language in submitted articles must be clear, correct, and unambiguous. Any typographical or grammatical errors should be corrected at revision, so please note any specific errors here.

Reviewer #1: Yes

Reviewer #2: No

**********

6. Review Comments to the Author

Please use the space provided to explain your answers to the questions above and, if applicable, provide comments about issues authors must address before this protocol can be accepted for publication. You may also include additional comments for the author, including concerns about research or publication ethics.

You may also provide optional suggestions and comments to authors that they might find helpful in planning their study.

(Please upload your review as an attachment if it exceeds 20,000 characters)

Reviewer #1: Clarity on the Validation of the MHCP-t: Data Tool: The manuscript's handling of the MHCP-t: Data tool's validation is inconsistent and potentially a conceptual flaw.

The Methods section states that content validity for the Data tool will only be assessed at the "scale level" using the second questionnaire. The Abstract and Objectives imply a full content validity assessment for "all three tools."

The justification that it "inventories sources rather than scalable constructs" (Line 63, 334) is a critical distinction. However, the protocol then proposes to assess its "overall content validity" (Line 335) using questions about relevance and comprehensiveness from Box 1.

Action Required: The authors must more clearly define what "content validity" means for an inventory tool versus an evaluative scale. Is the goal to ensure the list of data sources is comprehensive and relevant, rather than validating a set of scaled items? The distinction between "item-level" and "scale-level" validity for this tool should be explicitly stated and consistently applied throughout the manuscript, particularly in the Abstract, Objectives, and Data Analysis sections. The authors should consider if a different term, such as "utility" or "comprehensiveness," might be more accurate for the Data tool's assessment to avoid conflating it with the psychometric validation of the other two tools.

Feasibility of "Exhaustive Sampling": The manuscript proposes to recruit an "exhaustive sample" of 40-50 participants for each tool (Line 200), totaling 120-150 experts. This is an ambitious target in a specialized field within a single country.

Action Required: The authors should provide more detail on the expert pool in Tunisia. Are there existing registries, networks, or estimates of the total number of individuals who meet the inclusion criteria? Providing this context would strengthen the rationale for the sample size target and demonstrate its feasibility. If 40-50 truly represents the entire universe of eligible experts for each tool, this should be explicitly stated. If not, the term "exhaustive sampling" may be misleading, and a justification for why this number is adequate despite not being exhaustive should be provided.

Burden on Participants: The data collection process is substantial. Participants are asked to: 1) Complete their assigned MHCP-t (which itself is likely lengthy), 2) Complete an item-by-item validation survey for their tool, and 3) Complete a second global validation survey. This represents a significant time commitment.

Action Required: The manuscript would benefit from including an estimated completion time for each of the three MHCP-t tools and the accompanying surveys. This information is crucial for assessing the risk of respondent fatigue and low response rates, a limitation the authors themselves acknowledge. How will the study mitigate this burden beyond providing training? Will participants be compensated or incentivized for their time?

Reviewer #2: The manuscript presents an interesting and potentially valuable proposal for the development of the Migrant Health Country Profile tools (MHCP-t). However, the current submission describes a protocol and proposed validation process rather than findings derived from an implemented study. At present, the manuscript outlines the planned mixed-methods approach, including expert surveys and focus group discussions, but the tools themselves have not yet been tested in practice and no empirical results are available.

Given this, the manuscript is a research proposal or study protocol than as a completed research article. While the methodological design are promising, the contribution to the literature would be significantly strengthened once the proposed surveys and focus group discussions have been conducted and the results analyzed. Empirical evidence demonstrating the tools’ content validity, feasibility, and acceptability would allow readers to better assess the practical usefulness and methodological robustness of the MHCP-t.

I would therefore recommend considering the manuscript for publication after the study has been completed, including the implementation of the surveys with national experts and the focus group discussions. Presenting the resulting data and analysis alongside the methodological framework would provide a more substantial contribution to the field and allow for a clearer evaluation of the proposed tools’ effectiveness and applicability.

**********

7. PLOS authors have the option to publish the peer review history of their article (what does this mean?). If published, this will include your full peer review and any attached files.

If you choose “no”, your identity will remain anonymous but your review may still be made public.

**Do you want your identity to be public for this peer review?**  For information about this choice, including consent withdrawal, please see our Privacy Policy.

Reviewer #1: **Yes:** mohamed abdelkarim

Reviewer #2: No

**********

[NOTE: If reviewer comments were submitted as an attachment file, they will be attached to this email and accessible via the submission site. Please log into your account, locate the manuscript record, and check for the action link "View Attachments". If this link does not appear, there are no attachment files.]

To ensure your figures meet our technical requirements, please review our figure guidelines: https://journals.plos.org/plosone/s/figures

You may also use PLOS’s free figure tool, NAAS, to help you prepare publication quality figures: https://journals.plos.org/plosone/s/figures#loc-tools-for-figure-preparation.

NAAS will assess whether your figures meet our technical requirements by comparing each figure against our figure specifications.

# Author comment: Author response to Decision Letter 1

**DOI:** 10.1371/journal.pone.0352171.r002

8 May 2026

The Editorial Office

PLOS ONE

23 April 2026

Dear Dr Alamgir,

Thank you for the opportunity to revise our manuscript “Assessing the content validity of the Migrant Health Country Profile Tools in Tunisia: A mixed methods study protocol” (PONE-D-25-69071R1). In response to your valuable feedback, we have carefully examined the reviewers' and editors’ comments and have effectively addressed them as outlined in the response table below and in the marked and clean manuscript document.

Our response demonstrates our commitment to collaborating with PLOS ONE and aligning our manuscript with the journal’s high standards. We are happy to make any further changes, if required.

Thank you for your time, support, and consideration.

Sincerely,

Farah Seedat & Ana Requena Mendez

------

Assessing the content validity of the Migrant Health Country Profile Tools in Tunisia: A mixed methods study protocol

PONE-D-25-69071R1 manuscript resubmission: Response to reviewer comments

Reviewer 1

1 Clarity on the Validation of the MHCP-t: Data Tool: The manuscript's handling of the MHCP-t: Data tool's validation is inconsistent and potentially a conceptual flaw.

The Methods section states that content validity for the Data tool will only be assessed at the "scale level" using the second questionnaire. The Abstract and Objectives imply a full content validity assessment for "all three tools."

The justification that it "inventories sources rather than scalable constructs" (Line 63, 334) is a critical distinction. However, the protocol then proposes to assess its "overall content validity" (Line 335) using questions about relevance and comprehensiveness from Box 1.

Action Required: The authors must more clearly define what "content validity" means for an inventory tool versus an evaluative scale. Is the goal to ensure the list of data sources is comprehensive and relevant, rather than validating a set of scaled items? The distinction between "item-level" and "scale-level" validity for this tool should be explicitly stated and consistently applied throughout the manuscript, particularly in the Abstract, Objectives, and Data Analysis sections. The authors should consider if a different term, such as "utility" or "comprehensiveness," might be more accurate for the Data tool's assessment to avoid conflating it with the psychometric validation of the other two tools.

Response: Thank you for this important comment, which highlights the need for greater conceptual clarity in validating the MHCP t: Data tool. We agree that the distinction between validating an inventory tool versus an evaluative scale must be articulated more explicitly and applied consistently throughout the manuscript.

Indeed, the MHCP t: Data tool is designed as an inventory of existing data sources, not as an evaluative scale. Its individual entries correspond to external entities (e.g., registries, surveillance systems, and administrative datasets), and the primary purpose is to determine what these data sources cover, rather than to produce scaled items. Consequently, item-level content validity, which assesses whether individual items are relevant indicators of an underlying construct, is not conceptually applicable to the Data tool. Instead, validity for this tool is addressed at the tool level, focusing on whether the tool as a whole is relevant, comprehensive, and fit for the purpose of mapping the national data landscape for migrant health indicators. In this context, our use of the term “content validity” refers to the adequacy of coverage and relevance of the inventory, not to psychometric validation of individual item indicators. To avoid conflation with scale-based validation approaches, we now explicitly frame this assessment as tool-level validity, encompassing the relevance, comprehensiveness, feasibility, and acceptability of the entire tool.

In contrast, the MHCP t: Policy tool and relevant parts of the MHCP t: Provision tool operationalise defined constructs related to how well migrants are integrated into policy environments and service provision. Their items function as indicators of these constructs and therefore require item-level content validity assessment to ensure relevance, clarity, and comprehensive coverage of the underlying constructs.

To address this concern, we have revised the Abstract, Objectives, Methods, and Data Analysis sections to consistently state this, as below:

Abstract (page 3 lines 44-50):

“Two subsequent surveys will evaluate: 1) item-level content validity (relevance, comprehensiveness, and comprehensibility for individual items) for the MHCP-t: Policy and MHCP-t: Provision, where items function as indicators of the extent to which migrants are integrated into health policies and service provision and therefore require direct assessment at the item level, and 2) tool-level validity, feasibility, and acceptability for all three tools, including the MHCP-t: Data tool, where the primary objective is to assess the comprehensiveness and relevance of the inventory as a whole rather than the validity of individual items as indicators.”

We have also expanded on the explanation of the tools in the Abstract (page 3 lines 38-39) to make this distinction clear:

“Our transdisciplinary consortium co-developed the Migrant Health Country Profile tools (MHCP-t) – an innovative package of three survey tools that nationally map migrant health data sources (MHCP-t: Data tool), document migrant health policies and the integration of migrants in policies (MHCP-t: Policy tool), and the integration of migrants into healthcare provision (MHCP-t: Provision tool) for key diseases.”

Introduction:

Page 7 lines 124-128

“MHCP-t: Policy documents policies for migrant health … The tool comprises seven sections: … the integration of migrants into healthcare entitlement frameworks, and measures of policy implementation and change.

MHCP-t: Provision explores the integration of migrants into healthcare provision..”

Page 9 lines 164-172:

“This validation process will involve both item-level (question-level) content validity and tool-level (or scale-level) content validity. Specifically, item- and tool-level content validity will be assessed for the entitlement and measures of policy implementation and change sections of MHCP-t: Policy tool as well as for the complete MHCP-t: Provision tool. For the remaining sections of the MHCP-t: Policy tool and the complete MHCP-t: Data tool, content validity will be assessed only at the tool-level. These domains fundamentally differ as they serve primarily as mapping exercises rather than evaluative instruments.”

Objectives (page 9 lines 181-3):

“The primary objective of this study is to evaluate the content validity of the three Migrant Health Country Profile Tools: (1) MHCP-t: Data, (2) MHCP-t: Policy, and (3) MHCP-t: Provision. This will involve tool-level content validity of all three tools and item-level content validity for the entitlement and measures of policy implementation and change sections of the MHCP-t: Policy tool and the MHCP-t Provision tool.”

Methods

Pages 12-13 lines 246-275:

“Upon completing the relevant MHCP-t, participants of the MHCP-t: Policy tool and MHCP-t: Provision tool will encounter two structured validation questionnaires administered through the RedCap platform while participants of the MHCP-t: Data tool will only receive one. The first questionnaire focuses on item-specific content validity assessment, asking respondents to evaluate each question's quality across the dimensions of comprehensibility, relevance, and capturability. Using a 5-point Likert scale ranging from "Very low quality" (1) to "Exceptional quality" (5), participants will rate each item. Those providing ratings of 1 or 2 will be required to submit qualitative explanations for their low evaluations.

This granular validation approach will be applied uniformly throughout the entire MHCP-t: Provision tool and will not be applied for the MHCP-t: Data tool. For the MHCP-t: Policy tool, evaluation will be restricted to the measures of policy implementation and change and entitlement sections. Items within these sections as well as those in the MHCP-t: Provision tool function as indicators of the extent to which migrants are integrated into health policies and healthcare service provision and therefore require direct item-level assessment. The remaining sections of the MHCP-t: Policy tool together with the MHCP-t: Data tool serve primarily as mapping exercises rather than evaluative instruments; consequently item-level validity is not required for these sections.

The second questionnaire adopts a holistic approach, assessing tool-level content validity as well as feasibility, and acceptability across all three tools in the MHCP-t package. This comprehensive evaluation incorporates the standardised questions detailed in Box 1, allowing for systematic comparison across tool types and user groups. Three questions (3, 4, and 7) assess overall tool-level content validity of each tool, specifically addressing the relevance, comprehensiveness, and comprehensibility of each tool as a whole. This assessment is conducted for all three tools, including the MHCP-t: Data tool to determine whether the overall tool adequately captures the intended purpose. The remaining questions assess feasibility and acceptability. Respondents will be asked to rate each question on a 4-point Likert scale with the exception of question 4 which has a binary response of “yes” or “no”.”

Page 15 lines 295 and 308-309:

“The psychometric evaluation will employ three robust validation metrics to systematically assess the content validity of the items of the MHCP-t: Policy and relevant sections of the MHCP-t: Provision tools.

The second questionnaire (box 1) will be subjected to detailed descriptive statistical analysis for all three MHCP-t, including the MHCP-t: Data tool, which will only have this part of the analysis for the tool-level validity, feasibility, and acceptability.”

2 Feasibility of "Exhaustive Sampling": The manuscript proposes to recruit an "exhaustive sample" of 40-50 participants for each tool (Line 200), totaling 120-150 experts. This is an ambitious target in a specialized field within a single country.

Action Required: The authors should provide more detail on the expert pool in Tunisia. Are there existing registries, networks, or estimates of the total number of individuals who meet the inclusion criteria? Providing this context would strengthen the rationale for the sample size target and demonstrate its feasibility. If 40-50 truly represents the entire universe of eligible experts for each tool, this should be explicitly stated. If not, the term "exhaustive sampling" may be misleading, and a justification for why this number is adequate despite not being exhaustive should be provided.

Response: Thank you for noting the ambitious nature of the proposed sampling strategy. The target of 40–50 participants per tool was selected by balancing published recommendations for sample sizes in content validity studies (e.g., COSMIN guidelines) with the highly specialised scope of the topic. This approach was discussed extensively with the MHCP t Expert Working Group, who advised that recruiting more than 50 participants for any single tool would not be feasible within the national context.

As no official registries or comprehensive networks of eligible experts exist, we worked closely with the MHCP t Expert Working Group to identify and invite individuals who meet the inclusion criteria for each tool. As you correctly note, this approach did not allow us to know for certain the total number of eligible experts nationwide. We therefore agree that purposive sampling is a more accurate description than “exhaustive sampling.”

Purposive sampling is appropriate in this context, as content validity assessment requires the inclusion of individuals with specific, in depth expertise relevant to the constructs being evaluated. Our sampling strategy was designed to ensure sufficient diversity of expertise across policy, service provision, and data systems, aligning with established methodological guidance for expert based validation studies. Indeed, in Tunisia the completion of the tools so far includes 25 participants for the MHCP-t: Data, 29 participants for MHCP-t: Policy, and 40 participants for MHCP-t: Provision.

We have revised the manuscript accordingly to reflect this clarification.

Page 10 lines 205-211

“Guidelines for sample sizes for validation studies vary widely. For qualitative content validation, studies typically require 7-10 participants, however, data saturation is more important, whereas survey-based approaches generally necessitate 50-100 respondents to ensure adequate statistical power.[25] Balancing sample size requirements against the limited pool of individuals with appropriate expertise in migrant health policy and health system integration, we will employ purposive sampling to recruit approximately 40-50 eligible participants who meet the criteria outlined in Table 1.”

3 Burden on Participants: The data collection process is substantial. Participants are asked to: 1) Complete their assigned MHCP-t (which itself is likely lengthy), 2) Complete an item-by-item validation survey for their tool, and 3) Complete a second global validation survey. This represents a significant time commitment.

Action Required: The manuscript would benefit from including an estimated completion time for each of the three MHCP-t tools and the accompanying surveys. This information is crucial for assessing the risk of respondent fatigue and low response rates, a limitation the authors themselves acknowledge. How will the study mitigate this burden beyond providing training? Will participants be compensated or incentivized for their time?

Response: Thank you for highlighting the potential burden associated with the data collection process. Based on previous work in which 20 participants completed all three MHCP t tools over a total of nine hours, we anticipated that completion of a single MHCP t tool together with the associated item level and overall validation questionnaires would require a maximum of approximately five hours per participant. This estimate has been confirmed during the current phase of data collection.

To mitigate participant burden and reduce the risk of respondent fatigue, participants are able to complete the tool and validation questionnaires in stages, with the flexibility to return to them over time. In addition, participants are incentivised through the provision of a gift voucher upon completion of the tool. Members of the expert working group, who identified participants through their professional networks, also follow up individuals who had not yet completed the tool or surveys to identify any issues and to provide encouragement and support as needed.

We have incorporated this information into the manuscript (page 12, lines 236–243) to improve transparency regarding time commitment, burden mitigation strategies, and participant incentives:

“Based on the process evaluation, we anticipate that completion of one MHCP-t and the associated validation questionnaires will take approximately four hours in total; however, participants will be able to complete the tool and questionnaires in stages, with the flexibility to return to them over time. To encourage completion and reduce respondent burden and fatigue, participants will receive a gift voucher upon completion of the tool. In addition, as participants are contacts identified through members of the expert working group network, we will follow them up to monitor progress and to offer support as needed during completion of the tool and validation questionnaires.”

We have also included this information in the discussion section (page 19 line 371). To mitigate this, we will provide in-depth training and facilitate deep engagement with respondents, led by the credible leadership and members of the national working group, we will follow participants up, and compensate them with a gift voucher.

Reviewer 2

4 The manuscript presents an interesting and potentially valuable proposal for the development of the Migra

Attachment Submitted filename: MHCPt_Validation_PLOSONE_Response_Final.docx

# Editor report: Decision Letter 1

**DOI:** 10.1371/journal.pone.0352171.r003

**Authors:** AKM Alamgir

7 Jun 2026

Assessing the content validity of the Migrant Health Country Profile Tools in Tunisia: A mixed methods study protocol

PONE-D-25-69071R1

Dear Seedat,

We’re pleased to inform you that your manuscript has been judged scientifically suitable for publication and will be formally accepted for publication once it meets all outstanding technical requirements.

Within one week, you’ll receive an e-mail detailing the required amendments. When these have been addressed, you’ll receive a formal acceptance letter and your manuscript will be scheduled for publication.

An invoice will be generated when your article is formally accepted. Please note, if your institution has a publishing partnership with PLOS and your article meets the relevant criteria, all or part of your publication costs will be covered. Please make sure your user information is up-to-date by logging into Editorial Manager at Editorial Manager® and clicking the ‘Update My Information' link at the top of the page. For questions related to billing, please contact billing support.

If your institution or institutions have a press office, please notify them about your upcoming paper to help maximize its impact. If they’ll be preparing press materials, please inform our press team as soon as possible -- no later than 48 hours after receiving the formal acceptance. Your manuscript will remain under strict press embargo until 2 pm Eastern Time on the date of publication. For more information, please contact onepress@plos.org.

Kind regards,

AKM Alamgir, PhD

Academic Editor

PLOS One

Additional Editor Comments (optional):

Reviewers' comments:

# Editor report: Acceptance letter

**DOI:** 10.1371/journal.pone.0352171.r004

**Authors:** AKM Alamgir

PONE-D-25-69071R1

PLOS One

Dear Dr. Seedat,

I'm pleased to inform you that your manuscript has been deemed suitable for publication in PLOS One. Congratulations! Your manuscript is now being handed over to our production team.

At this stage, our production department will prepare your paper for publication. This includes ensuring the following:

* All references, tables, and figures are properly cited

* All relevant supporting information is included in the manuscript submission,

* There are no issues that prevent the paper from being properly typeset

You will receive further instructions from the production team, including instructions on how to review your proof when it is ready. Please keep in mind that we are working through a large volume of accepted articles, so please give us a few days to review your paper and let you know the next and final steps.

Lastly, if your institution or institutions have a press office, please let them know about your upcoming paper now to help maximize its impact. If they'll be preparing press materials, please inform our press team within the next 48 hours. Your manuscript will remain under strict press embargo until 2 pm Eastern Time on the date of publication. For more information, please contact onepress@plos.org.

You will receive an invoice from PLOS for your publication fee after your manuscript has reached the completed accept phase. If you receive an email requesting payment before acceptance or for any other service, this may be a phishing scheme. Learn how to identify phishing emails and protect your accounts at https://explore.plos.org/phishing.

If we can help with anything else, please email us at customercare@plos.org.

Thank you for submitting your work to PLOS ONE and supporting open access.

Kind regards,

PLOS ONE Editorial Office Staff

on behalf of

Dr AKM Alamgir

Academic Editor

PLOS One
